# Unlocking the Nutraceutical Potential of Legumes and Their By-Products: Paving the Way for the Circular Economy in the Agri-Food Industry

**DOI:** 10.3390/antiox13060636

**Published:** 2024-05-24

**Authors:** Fanghua Guo, Renan Danielski, Sarusha Santhiravel, Fereidoon Shahidi

**Affiliations:** 1 Department of Biochemistry, Memorial University of Newfoundland, St. John’s, NL A1C 5S7, Canada; fguo@mun.ca (F.G.); rdanielski@mun.ca (R.D.); ssanthiravel@mun.ca (S.S.); 2 State Key Laboratory of Food Science and Resources, Nanchang University, Nanchang 330047, China

**Keywords:** legume hulls, bioefficiency, polyphenols, bioactive peptides, biological activity

## Abstract

Legumes, including beans, peas, chickpeas, and lentils, are cultivated worldwide and serve as important components of a balanced and nutritious diet. Each legume variety contains unique levels of protein, starch, fiber, lipids, minerals, and vitamins, with potential applications in various industries. By-products such as hulls, rich in bioactive compounds, offer promise for value-added utilization and health-focused product development. Various extraction methods are employed to enhance protein extraction rates from legume by-products, finding applications in various foods such as meat analogs, breads, and desserts. Moreover, essential fatty acids, carotenoids, tocols, and polyphenols are abundant in several residual fractions from legumes. These bioactive classes are linked to reduced incidence of cardiovascular diseases, chronic inflammation, some cancers, obesity, and type 2 diabetes, among other relevant health conditions. The present contribution provides a comprehensive review of the nutritional and bioactive composition of major legumes and their by-products. Additionally, the bioaccessibility and bioavailability aspects of legume consumption, as well as in vitro and in vivo evidence of their health effects are addressed.

## 1. Introduction

Legumes belong to the *Leguminosae* family, which includes oil-producing crops such as peanuts (*Arachis hypogaea*) and soybeans (*Glycine max*). The latter is the fifth largest food crop after wheat, rice, corn, and barley [1]. The word “pulse” is derived from the Latin word “puls or pultis” meaning thick slurry, and according to the Food and Agriculture Organization (FAO) of the United Nations, pulses include beans, broad beans, peas, chickpeas, cowpeas, pigeon peas, lentils, bambara beans, vetches, lupins, and other “minor” pulses, but does not include peanuts and soybeans [2], all of which are legumes. Beans, peas, chickpeas, and lentils are the most commonly consumed legumes, while peanuts and soybeans are primarily used for oil extraction. Pulses are widely cultivated around the world and have a long history of food use. Bean seeds are mainly composed of embryos (cotyledons and hypocotyl) and the seed coat. The cotyledons and hypocotyls are rich in protein and carbohydrates, while the seed coat mainly contains biologically active substances such as flavonoids and other phenolic compounds. In addition, legumes are also rich in micronutrients such as iron, zinc, and vitamins. 

With the changes in dietary patterns in recent years, the incidence of various chronic diseases has significantly increased, leading individuals to gradually seek foods with superior nutritional quality, including legumes. As good sources of amino acids, legumes can be used to complement cereals in terms of amino acid balance [3]. Legumes are also rich in dietary fiber and have a low glycemic index (GI), meaning that after consumption, these foods do not cause high fluctuations in plasma glucose levels, thus serving as an important tool in blood sugar control [2,4,5]. In addition, studies have found that the daily intake of a portion of legumes is related to positive effects in lowering blood lipids [3], controlling obesity [4,6], treating colitis [7], and preventing cardiovascular and cerebrovascular diseases [8], breast cancer [9], type 2 diabetes [10], and non-alcoholic fatty liver disease [11]. Although dietary intake of legumes exhibits various health benefits, their per capita consumption is low, at only 22 g/person/day, especially in Central Asia and the Caucasus [12]. 

Protein is one of the most important nutrients, and is derived mainly from meat, poultry, fish, eggs, nuts, legumes, and milk. As the global population continues to increase, the demand for protein has led to higher meat consumption, resulting in a significant environmental burden due to the large greenhouse gas emission and utilization of water associated with meat production [13]. In contrast, legumes have lower greenhouse gas emissions compared to meat and dairy products [12]. Furthermore, legumes take part in symbiotic nitrogen-fixing with cyanobacteria, leading to soil enrichment. Therefore, they are a good source of sustainable protein. However, the consumption of meat has reached 112 g/person/day [12], replacing legumes as the main source of protein. In light of their high yield fluctuations, low profitability, and reduced investment, pulses have gradually lost their competitive advantage [12,14]. Therefore, returning pulses to an important position in human diet and agriculture is an important step towards achieving a green and sustainable agricultural food system. 

Legumes are rich in a myriad of health-promoting molecules. These compounds are not only present in their edible portions but are also part of their residual fractions. Repurposing legume by-products through a health-promoting prism can open new possibilities for their value-added applications beyond traditional use, resulting in an increased number of wholesome dietary choices for consumers, while also helping farmers and the food industry build new production systems and business opportunities. In selected geographical areas, pulse by-products may include the seed coat, which is its outer shell. It has a hard texture, dark color, and poor taste, thus seriously affecting pulses’ quality and market acceptance. Therefore, dehulling is the main step in the industrial processing of pulses [15]. Pulse hulls account for approximately 8–20% of the whole seed and are pulses’ primary by-product; however, broken cotyledons and hypocotyls are also produced [16]. In 2022, global pulse production was 93 million metric tons (MMT), resulting in large amounts of processing by-products that are often discarded or used as low-value feed [17]. The output of soybeans (not included in pulses) and peanuts in 2022 was about 861 and 49 MMT, respectively [18]. After oil extraction, a large amount of soybean and peanut meal is produced. The downcycling of these by-products causes economic losses and possible environmental pollution.

The upcycling of food processing by-products is an important part of sustainable development. Some efforts have been concentrated towards this goal, with a few successful examples, such as those using vegetable waste and potato peel [19]. Pulse hulls are rich in polyphenols and dietary fiber, thus rendering great potential in the prevention and treatment of oxidative stress, chronic inflammation, and non-alcoholic fatty liver disease [20,21,22,23,24,25,26]. The health benefits of legume by-products can serve as the basis for the development of functional food ingredients and nutraceuticals. In addition, protein from soybean and peanut meals also has a significant economic value [27,28]. This review compiles the nutritional value and bioactive compounds of legume by-products, with a further discussion on their potential health benefits and prospective applications that may pave the way for their value-added utilization and sustainable developments. 

## 2. Legumes and Their By-Products

**Figure 1:**
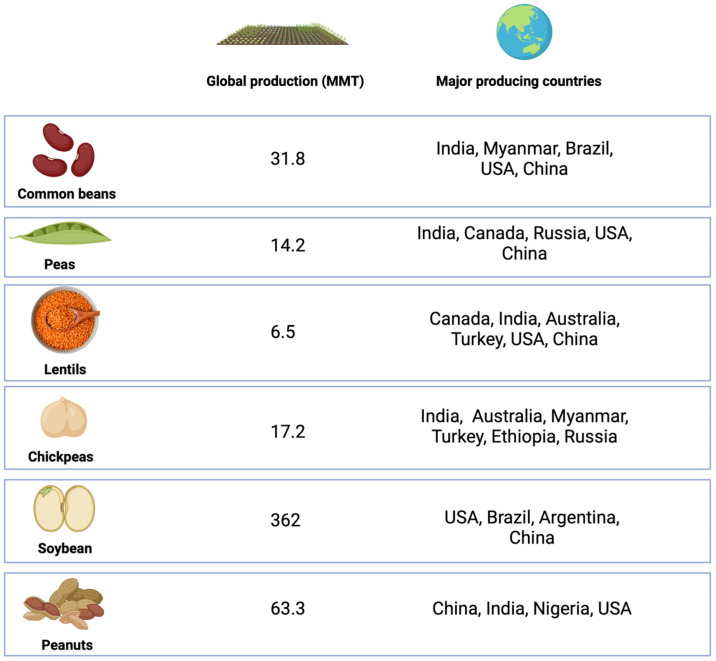
Yield and main producing countries for the major legumes cultivated in the world. Created with Bio Render.

Common beans, peas, lentils, chickpeas, soybean, and peanuts are among the main legumes produced in the world (Figure 1).

### 2.1. Beans 

Common beans (*Phaseolus vulgaris* L.), also known as French bean, kidney bean, snap bean, runner bean, or string bean, are grown well in areas with moderate rainfall [29]. Beans were first domesticated in the Americas and Asia more than 3000 years ago, and may have originated in Central America [30]. The main producing countries of kidney beans are India, Myanmar, Brazil, United States, and China, with the global production in 2018 reaching approximately 31.8 million tons [12]. Common bean is rich in protein and starch, accounting for 20.9–27.8% and 41.5% of the dry weight of the seeds, respectively, with low oil content (0.9–2.4%) and about 10% dietary fiber (mainly in the bean hulls) [31]. Beans are rich in amino acids and have higher levels of phenylalanine than peas and lentils [32]. In addition, common beans are also rich in macro and trace elements, such as iron (4.2–7.8 mg/100 g), zinc (2.1–3.8 mg/100 g), and calcium (88.1–162 mg/100 g), with contents varying according to the variety [33]. Antinutrients (oligosaccharides, trypsin inhibitors, α-amylase, tannins, phytates) are also present in common beans, but usually removed (except phytates) during the cooking process [12]. 

The by-products of common bean processing are mainly the hulls with some broken cotyledons and hypocotyls (usually powder), as well as abnormally colored or shriveled seeds. Common bean hulls are rich in phenolic compounds (e.g., anthocyanins, procyanidins, flavonols, phenolic acids) and dietary fiber (see Section 3). The nutritional profile and bioactive molecules in beans are the basis for their health benefits, such as antioxidant, anti-inflammatory, anti-obesity, anti-diabetic, and cardioprotective effects, as well as their application as raw materials in food [34].

### 2.2. Peas

Peas (*Pisum sativum* Linn.), which originated in the Middle East more than 8000 years ago, are the second most consumed legume crops [35]. Common peas can be divided into yellow, brown, and green peas according to their color, also differing in particle size and skin thickness. Peas are widely cultivated in several countries, including Canada, India, Russia, United States, and China, due to easy mechanical harvesting, easy rotation among grains, resistance to low temperatures, and excellent nitrogen-fixing ability due to their symbiosis with cyanobacteria [36]. In 2019, the area under cultivation was 7,166,876 hectares (Ha), with production accounting for 14,184,249 tons, and India being the largest producer [37]. In Europe and North America, peas are mainly consumed in the diet, with their residual fractions used to produce feed mixed with ground grains; in Asia and South America, peas are used mainly for human consumption [36].

Common pea is rich in protein (18.3–31%) and starch (45%), and is also a good source of energy. Dietary fiber accounts for about 12%, while the oil content of peas is low (0.6–5.5%) [31]. Peas are rich in lysine [32] and minerals, including iron (2.2–9 mg/100 g), calcium (46–157 mg/100 g), and zinc (1.7–6.4 mg/100 g) [33]. Antinutritional factors in peas include α-galactosides, trypsin inhibitors, chymotrypsin inhibitors, lectins, tannins, and phytates. These are substances naturally found in plant material that can interfere with nutrient absorption and bioavailability in the body [12]. In recent years, in-depth research on pea starch, protein, and dietary fiber has been conducted, and their application in food, textile, light chemical, pharmaceutical, and other industries has been further developed [29,38]. Pea protein has good functional properties, acting as an emulsifier and gelling agent, and can be used as a food additive in the processing of cereals, dairy, and meat products to improve product quality and structure. 

The physiological effects of peas have also been extensively studied. Pea protein hydrolysate can inhibit the secretion of proinflammatory factors such as nitric oxide (NO), tumor necrosis factor-α (TNF-α), and interleukin-6 (IL-6) [39,40]. Peas are rich in dietary fiber, promoting a positive balance of the intestinal flora and reducing the risk of colon cancer [41,42]. Dietary fiber is mainly found in the hulls, which are a by-product of pea processing and make up 8–11% of the weight of the seeds. Meanwhile, pea shells are also rich in phytochemicals, such as phenols, including tocopherols, terpenes, and carotenoids, with polyphenols predominating as the major bioactive compounds. Most of the phenolic substances in pea seeds are concentrated in the hulls. Moreover, dark-colored seeds have a higher polyphenol content than light-colored ones. Some polyphenols in these by-products can be absorbed and metabolized, and play a key physiological role [20,21,23,24,25]. Most pea hulls are disposed of or used as fertilizer or animal feed. In recent years, these residual portions have also been used to produce fiber. 

### 2.3. Lentils

Lentil (*Lens culinaris*) is believed to have originated in the Near East and existed for more than 8000 years [43]. It can be round, oval, or heart-shaped. According to the color of the hull and cotyledon, lentils can be divided into brown, green, black, red, yellow, and spotted green varieties, with red and green being the predominant types in the market. This crop is resistant to drought and low temperatures, being widely cultivated around the world, with production increasing from 3.05 million tons in 1999 to 6.5 million tons in 2018. Canada, India, Australia, Turkey, United States, and China are the main lentil-producing countries [12]. 

Consumption of lentils is associated with reduced risk of dyslipidemia, colon cancer, and type 2 diabetes [44], owing to their rich composition of macronutrients and minor compounds. Lentils are a source of high-quality protein (23–32%) [31], including all the essential amino acids, especially lysine [32], being a great complimentary food together with cereals in order to achieve an ideal amino acid balance. Lentils are high in fiber (about 12%) [31], which helps maintain a healthy digestive tract, and low in fat (0.8–2%) [31], thus reducing the body’s total caloric intake. Lentils contain up to 46% starch [31], and are gluten-free and a nutritional alternative to wheat products for people with celiac disease [45]. In addition, lentils provide essential vitamins and minerals [46]. Lentils are lower in antinutritional factors than other legumes, mainly trypsin inhibitors and tannins [12]. Lentil polyphenols and dietary fiber are mainly located in their residual fractions, accounting for about 10% of the hull. The composition, metabolism, and activity of lentil hull polyphenols have been extensively studied [22,47,48]. The health benefits and use of lentil hull dietary fiber in food are constantly being explored. Therefore, lentil hulls hold great promise as value-added ingredients in different food applications.

### 2.4. Other Legumes

Chickpeas (*Cicer arietinum*) are the fourth largest legume crop after soybeans, peanuts, and beans, with an output of 17.2 million tons in 2018, and the major producing areas include India, Australia, Myanmar, Turkey, Ethiopia, and Russia [12]. Chickpeas have been cultivated for more than 5500 years and mainly include Kabuli (large seeds, light color) and Desi (small seeds, dark color) varieties [29]. The protein, starch, and fiber contents of chickpeas are 15.5–28.2, 44.4, and 9%, respectively, while the oil content (3.1–7%) is higher than that of lentils and beans [31]. Chickpeas are rich in tryptophan [32] and minerals, such as iron (3.4–5 mg/100 g), calcium (57.5–109 mg/100 g), and zinc (2.6–4.9 mg/100 g) [33]. Their antinutritional factors include oligosaccharides, protease inhibitors, saponins, and phytates [12]. Chickpea hulls, a processing by-product, are rich in dietary fiber and polyphenols. In addition, hulls also contain a small amount of protein (7.3 g/100 g), starch (0.2–0.5 g/100 g), and oil (1.6 g/100 g). The dietary intake of chickpeas has many health benefits related to cardiovascular disease, type 2 diabetes, and some cancer, among other conditions [49,50]. Meanwhile, chickpeas are also used as ingredients in the production of bread and snacks [51].

Soybean (*Glycine max*) is the largest legume crop with a global production of 362 million tons in 2018, and major producing countries include the United States, Brazil, Argentina, and China [12]. Soybeans are richer in oil (17.7–21%) when compared with other legumes [31], making them an important oil crop. In addition, soybean is also rich in protein and has a balanced amino acid profile [52]. The amount of polyunsaturated fatty acids, which are recognized as having a positive effect on health, in soy is 11.225 mg/100 g, higher than in other pulses [52]. In addition to being used for oil extraction, soybeans are also widely made into various soy products, such as tofu, soy milk, soy sauce, tempeh, natto, sufu, and meat analogues (in the form of textured vegetable protein) [52]. Dietary intake of soy is associated with health benefits such as cardiovascular and metabolic protection, anticancer properties, amelioration of menopause and osteoporosis symptoms, and maintenance of intestinal flora; however, the presence of soy isoflavones is controversial as it may cause thyroid disorders, sex hormone imbalance, and carcinogenesis [52]. Soybean meal, the fraction remaining after oil extraction, is the main by-product of soybean processing. This fraction is rich in protein and is mainly used as animal feed [28]. However, soybean meal contains several antinutritional factors, including trypsin inhibitors, phytates, and oligosaccharides, which can be reduced in activity or removed by cooking or fermentation [53,54].

Peanut (*Arachis hypogaea*) is the second largest legume crop after soybeans, with a global production of 63.3 million tons in 2018, and major producing countries are China, India, Nigeria, and the United States [12]. Peanuts originated in South America [12], and are rich in protein (25.8 g/100 g) and fat (49.24 g/100 g), especially unsaturated fatty acids (15.558 mg/100 g) [52]. Peanuts are low in antinutritional factors such as trypsin inhibitors, α-amylase, tannins, and phytates [12]. It is important to note that peanuts can be easily contaminated with aflatoxin, which is carcinogenic and difficult to remove [12]. Moreover, peanut allergy is common and is related to genetics, population, and diet, among other factors. Peanuts can be used for oil extraction or direct consumption. They can also be processed into products such as peanut butter. Similar to soybean meal, peanut meal contains protein and some fiber, and is generally used as animal feed.

## 3. Nutritional Profile and Bioactive Compounds in Legumes and Their By-Products

### 3.1. Proteins and Essential Amino Acids

Legume by-products are usually low in protein (less than 10%), where cotyledon and cotyl lost during dehulling are the main protein sources in this type of residual material [55]. Protein is the main component of peanut meal (40–50%) and is usually used as animal feed [27]. Recently, the value of these proteins has been investigated for further use. The traditional extraction methods of peanut protein include isoelectric precipitation, alcohol precipitation, isoelectric precipitation combined with alcohol precipitation, hot water extraction, and alkali solution isoelectric precipitation, which can improve the extraction rate in combination with enzymes, ultrafine grinding, radiation, microwave, and ultrasonic treatments [56]. Peanut protein has good water and oil binding capacity, emulsification capacity, foam capacity, and gel-forming properties. Meanwhile, peanut protein can be modified by high temperature, high pressure, alkaline conditions, phosphorylation, acylation, and enzymatic treatment [56]. Peanut protein can be used in food products such as meat analogs, breads, soups, candies, frozen desserts, and cakes [57]. It also has the potential for use in industrial applications such as protein membranes [58]. In addition, peanut proteolytic peptides have received widespread attention because of their many health benefits, such as antioxidant [59] and antihypertensive [60] properties. However, more clinical research is needed to explore their other potential health effects.

The protein content of soybean meal is around 40–49% and is high-quality protein, rich in essential amino acids, and one of the few plant proteins that can replace animal protein [28]. Globulin and albumin are the major proteins in soybean meal, accounting for approximately 90 and 5% of the content, respectively. These proteins have good emulsification, hydration, oil absorption, gel-forming, foaming, and conjunctival capacities, which supports their wide application as functional ingredients. They have been extensively studied from extraction and isolation to application, including the characterization and application of hydrolysates, in the food industry, as bio-based material and in biomedical areas [61,62,63,64,65].

Additionally, selected legumes are sources of several essential amino acids (EAAs). These nine amino acids (histidine, isoleucine, leucine, lysine, methionine, phenylalanine, threonine, tryptophan, and valine) cannot be synthesized in the body, although they are crucial for a number of biological processes. Therefore, the oral consumption of EAA-rich sources is the only route to obtain them. EAAs are involved in the production of red and white blood cells, nerve cell protection, muscle protein and growth hormones synthesis, mineral absorption and metabolism, and production of enzymes and antibodies, among other functions [66]. 

Soybean and chickpea contain all EAAs. On the other hand, pea and fava bean are limited in their tryptophan (the only EAA not present in these sources). Besides not containing tryptophan, lentil is also limited in its lysine. Among major legume sources, pea usually caries low amounts of EAAs, ranging from 0.18 (methionine) to 1% (leucine and lysine), while fava bean generally contains very high EAA contents, varying between 2.18 (methionine) and 14.5% (leucine) [67].

### 3.2. Dietary Fiber

Dietary fiber is an undigestible type of carbohydrate polymer that cannot be absorbed by the human small intestine but is of health significance due to its regulation of the colonic environment. Dietary fibers occur naturally in plant material or are extracted/synthesized, with a degree of polymerization (DP) of ≥3, including soluble and insoluble types. The main component of legume hulls is dietary fiber, with a content of more than 70%, which includes less than 10% soluble fiber [32,55]. Soybean meal and peanut meal also contain less than 10% dietary fiber [27,28]. Dietary fiber has the effect of regulating blood sugar and blood pressure, and lowering blood lipids. Studies have found that consuming pea fiber can regulate blood sugar levels, cholesterol, and phospholipid metabolism [68,69]. Ingesting a certain amount of dietary fiber can help promote intestinal peristalsis, remove toxic and harmful substances from the intestines, as well as promote the growth of probiotic microorganisms in the intestines. Dietary fiber is an important carrier of polyphenols. Dietary fiber entering the lower digestive tract is fermented by specific microorganisms, releasing bound phenolic compounds and minerals, while reducing colon pH, promoting the absorption of these nutrients and producing short-chain fatty acids, thereby supporting probiotics, including the growth of intestinal flora and maintaining the health of intestinal mucosa [70]. 

Studies have shown that the intake of dietary fiber can reduce the risk of heart disease, diabetes, obesity, and some cancers [71]. In addition to these health benefits, dietary fiber also has many functional properties, such as water retention, oil retention, and expansibility, and is used in food processing, such as the use of pea hull dietary fiber in beef patties to improve water retention [72]. In addition, it can be added to baked products such as bread to increase dietary fiber content [73]. Currently, there are several types of fiber made from pea and lupine hulls on the market, but other dietary fibers of pulse hulls also need to be developed and applied. Attention should also be paid to the safety of these fibers, such as their association with antinutritional factors such as alkaloids and phosphoprotease, as well as promotion of allergies [16].

### 3.3. Soluble Carbohydrates

Although carbohydrate content varies according to the legume type, ranging from insignificant amounts in *Glycine max* to half of the dry seed weight in certain pea types, the content of carbohydrates in legumes is usually around 33–46% [74]. It has been reported that legumes contain around 30 different soluble carbohydrates. Among these, sucrose is the main soluble carbohydrate, followed by the raffinose family of oligosaccharide, including raffinose, stachyose, and verbascose [75]. 

Although legume oligosaccharides can modulate gut microbiota by acting as prebiotics, they are associated with some level of intestinal discomfort following legume consumption, such as bloating and flatulence. This effect stems from the fact that the human gastrointestinal tract cannot digest galactosidase-containing oligosaccharides such as raffinose and stachyose due to the lack of α-galactosidase enzyme, which is responsible for cleaving the α-1,6 galactose linkage in these oligosaccharides. As such, the accumulation of undigested carbohydrates in the colon contributes to excessive fermentation promoted by the colonic bacteria, resulting in the production of gases such as carbon dioxide, methane, and hydrogen. These gases are responsible for the intestinal side effects linked to the intake of certain legumes [76].

### 3.4. Tocopherols and Tocotrienols 

Vitamin E refers to a group of fat-soluble vitamins, which includes four tocopherols and four tocotrienols. Tocopherol structure comprises a saturated phytyl residue at the C2 position, while tocotrienols have three double bonds in their phytyl group. Each subgroup has four different forms, namely α-, β-, γ-, and δ-, according to their number and pattern of methylation [77]. Vitamin E is known for its antioxidant properties and plays a crucial role in protecting cells from oxidative damage. Biological activities vary among the different forms of tocopherol and tocotrienol, with α-tocopherol being the most biologically active form in humans [78]. Legumes have the ability to synthesize tocopherols and tocotrienols, where oily fractions of certain legumes, particularly soybeans, are the major source. Moreover, α- and γ-tocopherols are the predominant forms of tocopherols found in seed oils [79]. The γ- tocopherol homolog is the major form present in the lentils cultivated in Canada, followed by α- and δ-tocopherols [80].

As evidenced in previous studies, soybeans have the highest content of tocopherol (90.40–120.96 μg/g) compared to other legumes. Moreover, it has been found that γ-tocopherol is the predominant type of tocopherol present in soybeans, lentil, chickpea, broad bean, lablab bean (*Lablab purpureus*), kulthi bean (*Macrotyloma uniflorum*), common beans, pea, and three lupin species, followed by δ- and α-tocopherols. More than 10 mg of total tocopherol per 100 g seeds is also present in pea, chickpea, soybean, and white lupin [81,82,83]. Furthermore, Górnaś et al. [84] investigated the tocopherol and tocotrienol contents of nine uncommon species belonging to the Fabaceae family, naturally grown in India. Although all four tocopherol forms (α-, β-, γ-, and δ-), along with α-, β-, and γ-tocotrienol were present in all the species analyzed, α- and γ-tocopherols and tocotrienol homologues were predominant. 

### 3.5. Carotenoids 

Carotenoids are a group of naturally occurring pigments, belonging to the tetraterpene class of compounds, with yellow, red, orange, and purple colors. These pigments are widely distributed in plants, algae, animals, photosynthetic bacteria, and some species of fungi and archaea. Isoprene units are the basic building blocks of carotenoids with a 40-carbon unit backbone [85]. Carotenoids are primarily classified as carotenes or xanthophylls. Examples of carotenes include α-, β-, and γ-carotenes, as well as lycopene, while xanthophylls include lutein, astaxanthin, zeaxanthin, canthaxanthin, β-cryptoxanthin, fucoxanthin, and peridinin. Around 50 carotenes and 800 xanthophylls have been reported in the literature [86]. Carotenoids carry physiological relevance due to their provitamin A activity, antioxidant activity, and bioactivities such as their role in preventing chronic degenerative diseases [87]. 

Although legumes present a lower level of carotenoids when compared to some fruits and vegetables, they still have several types of these pigments. Lutein, β-carotene, and cryptoxanthin are predominant carotenoids in legumes [88]. A study of the carotenoid composition of chickpeas, faba beans, dry beans, and lentils from the Mediterranean region revealed that all the tested samples contained lutein and β-carotene, with the former showing the highest abundance. Moreover, zeaxanthin was present in chickpeas, dry beans, and lentils; with the exception of dry beans, all of the samples contained violaxanthin and neoxanthin [89].

Zhang et al. [80] investigated the chemical composition of 20 lentil cultivars (10 red and 10 green) and demonstrated that the major carotenoid present was *trans*-lutein (64–78%), followed by *trans*-zeaxanthin (5–13%), with some isomeric forms also detected. Similar results were observed in another study [82] where the carotenoid content of 29 legume species was analyzed. It was found that the major carotenoids present in all samples were *trans*-lutein and *trans*-zeaxanthin. Among all samples, red kidney beans displayed the highest carotenoid content, followed by cowpeas and black soybeans, while white kidney beans and mung beans showed the lowest content [82].

### 3.6. Phytic Acid, Trypsin Inhibitors, and Saponins 

As noted already, legumes contain some antinutritional factors such as phytic acid, trypsin inhibitors, and saponins. As such, there are major limitations to the utilization of legumes at household and industrial levels as they hinder the digestibility and absorption of certain nutrients [90]. Phytic acid is the major storage form of phosphorous in legumes and its content ranges from 0.2 to 2.9%. Phytic acids can chelate micronutrients such as zinc and limit their bioavailability in the human body due to the lack of phytase enzymes in the digestive tract [91]. 

Trypsin inhibitors are mainly present in the cotyledons of legumes, which can reduce the digestion and absorption of dietary proteins. Depending on the molecular weight, trypsin inhibitors in legumes can be divided into two families, namely Kunitz (20 kDa) and Bowman–Birk (8 kDa). Most legumes contain either Kunitz or Bowman–Birk, except for soybean, which contains both. Trypsin inhibitors in lentils and common beans belong to the Bowman–Birk family [54]. 

Saponins are secondary compounds found in a variety of legumes, particularly lupins, lentils, chickpeas, peas, and beans. Saponin structure comprises a triterpene or steroid moiety (aglycone) linked to one or more sugar units (glycone) via glycosidic linkages. Saponins are responsible for the astringency and bitter taste of foods containing them [92]. 

### 3.7. Essential Fatty Acids 

Most legumes are low in lipids and contain no cholesterol. However, they are usually high in phytosterols. For instance, common beans showed a total sterol content of 5136 mg/100 g of oil, predominantly ΔS-stigmasterol (1351 mg/100 g oil), followed by campesterol (310 mg/100 g oil). Soybean has a considerably lower content of total sterol (350 mg/100 g oil), with β-sitosterol (186 mg/100 g oil) and campesterol (64 mg/100 g oil) being the major ones [81]. Furthermore, certain legumes have a high oil content, such as soybeans (47%), peanuts (45%), and chickpeas (15%). Their oil composition consists of mono- and polyunsaturated fatty acids (66.1–85.3%), with a low content of saturated fatty acids. The major fatty acids present in legumes are essential fatty acids linoleic (C18:2, omega-6) and α-linolenic (C18:3, omega-3) acids, ranging from 47.3% in orange lentils to 71.0% in black beans, as well as oleic, stearic, and palmitic acids in lesser amounts. Although necessary for several biological functions, these fatty acids cannot be synthesized by the body and can only be obtained from the diet [93]. 

Achieving the ideal balance between omega-3 (ω-3) and omega-6 (ω-6) fatty acids is crucial as they play different roles in the body. In general, a lower ratio of ω-6 to ω-3 (5:1) is considered ideal in terms of health benefits [94]. Legumes such as kidney, black, and navy beans have a low ratio (<1) of ω-6/ω-3 fatty acids. Similarly, mung bean, cowpea, and adzuki beans also have relatively lower ratios of omega-6 to omega-3. However, certain legumes, such as faba beans and chickpeas, have high ω-6/ω-3 ratios (14.59 and 19.67, respectively) [93]. Therefore, legumes are good sources of essential fatty acids, and in order to achieve a desirable ω-6/ω-3 balance, the combination of multiple legume sources is ideal.

### 3.8. Phenolic Compounds 

Phenolic compounds are plant secondary metabolites with the primary function of providing protection to these organisms against abiotic stress, including ultraviolet radiation, temperature extremes, reactive oxygen species (ROS), and pathogen attack. As a defense mechanism, phenolic compounds usually accumulate on the outer layers of plant material due to the exposure to herbivores, as well as on the surface of seeds in order to safeguard their reproductive process. In legumes, these substances are abundantly present in the seed coat (especially flavonoids) and in the cotyledons. This extremely diverse group of bioactives includes over 8000 identified molecules, which can be subdivided into several categories according to their structural similarities. The basic phenolic chemical structure contains one (monophenols) or more (polyphenols) hydroxyl groups attached to one or more aromatic rings with different side chains. Tocopherols and tocotrienols, usually referred to as vitamin E, are well-studied and widely used monophenols. However, most phenolic compounds found in nature are polyphenols. As such, some authors use the terms phenolic compound and polyphenol interchangeably [95].

The aromatic ring from phenolics’ basic unit comes from their precursors, the amino acids phenylalanine and, to a lesser extent, tyrosine. These are synthesized from shikimic acid, which also produces gallic acid through an alternative route. Gallic acid is the most abundant phenolic acid in nature. It can polymerize into gallotannins, a hydrolysable tannin subclass. The same can occur with ellagic acid, gallic acid’s dimeric form, which polymerizes into ellagitannins, another hydrolysable tannin subgroup. Through a series of enzyme-catalyzed reactions, phenylalanine is transformed into cinnamic acid, which in turn generates other phenolic acids. For instance, the enzymatic hydroxylation of cinnamic acid affords *p*-coumaric acid, which is the precursor for other phenolic groups (coumarins, stilbenes, and lignans). *p*-Coumaric acid can also enter the malonic acid pathway in its *p*-coumaroyl-COA form, ultimately generating several flavonoid subclasses, such as flavonols, isoflovonoids, anthocyanins, and flavan-3-ols through a cascade of enzymatic transformation. Catechin and its isomer epicatechin are the most representative compounds in the flavan-3-ol subgroup. They are able to polymerize into proanthocyanidins, one of the most structurally complex polyphenol classes in nature [96].

Phenolic acids, flavonoids, and tannins are the most relevant phenolic classes in legumes. Phenolic acids can be either hydroxybenzoic acid or hydroxycinnamic acid derivatives. The former possesses a C6-C1 backbone and includes gallic, vanillic, protocatechuic, syringic, and *p*-hydroxybenzoic acids. Legume seeds are generally rich sources of gallic and protocatechuic acids. Conversely, hydroxycinnamic acids have a C6-C3 backbone and include caffeic, *p*-coumaric, ferulic, sinapic, and chlorogenic acids among their most significant compounds [95]. Table 1 shows the distribution of phenolic acids and polyphenols in several legume by-products.

Flavonoids (C6-C3-C6) are the largest phenolic group in nature. As such, they can be categorized into a number of subclasses (flavonols, flavan-3-ols, flavanones, flavones, isoflavones, flavanonols, and anthocyanins) based on their hydroxylation and methylation patterns. Flavonoids’ basic unit consists of two aromatic rings connected to a central heterocyclic ring via a three-carbon bridge. Anthocyanins (anthocyanidins bound to a sugar molecule) are the only pigmented polyphenols. Their color can vary (red, purple, blue) according to structural changes induced by pH variations, and they are found in dark-colored legumes, such as black beans, lentils, and black soybeans [96].

Tannins are oligomeric and polymeric phenolics classified either as hydrolysable tannins or condensed tannins; the latter are also known as proanthocyanidins. The former are subdivided into gallotannins (repeating units of gallic acid) and ellagitannins (repeating units of ellagic acid), while catechin and epicatechin monomers are the building blocks of proanthocyanidins [95]. In legumes, tannins usually concentrate on the outer seed coat, being key players in oxidative stress mitigation. Lentils are excellent sources of procyanidins, a type of proanthocyanidin.

The distribution of phenolic compounds in their original sources is complex and such molecules can occur in different forms. Free phenolics are not bound to other matrix components and are readily soluble when mixed with an appropriate solvent. This characteristic also impacts their bioaccessibility and bioavailability, as phenolic compounds in the free form are usually readily digested in the gastrointestinal tract and able to cross the epithelial membrane of the small intestine, followed by a series of metabolization processes [95]. However, other types of soluble phenolics also exist, including the ones esterified to fatty acids and other organic acids, as well as phenolics attached to sugar moieties (glycosylated). As an example, Yang et al. [101] found several flavonoid glycosides, such as catechin-*O*-hexoside, kaempferol-*O*-hexoside, vixetin-*O*-rhamnoside, and taxifolin-*O*-hexoside, as part of the phenolic composition of nine mung bean varieties grown in Sri Lanka.

Contrary to free phenolics, the soluble-bound ones need to first be released from the molecules to which they are attached. The same can be said about insoluble-bound phenolics, which are covalently bound to cell wall components (e.g., cellulose, pectin, structural protein). As a consequence, such phenolic compounds generally present low bioaccessibility and bioavailability, being released only in the large intestine by the action of colonic bacteria. Such a feature is extremely relevant for certain types of source material, including legumes, where insoluble-bound phenolics can surpass 60% of all phenolics present [102]. For instance, in Chinese cultivars of mung bean, syringic, caffeic, *p*-coumaric, and ferulic acids were found in the insoluble-bound form and the sum of their contents represented 89.8% of all phenolic acids identified in the material [103]. Due to its relevance in terms of physiological impact, the bioefficiency of legume polyphenols will be further discussed in greater detail in Section 4. 

Phenolic compounds may serve as antioxidant substances that can act via multiple mechanisms. They can function as primary antioxidants by scavenging free radicals. This happens by the donation of hydrogen atoms derived from their hydroxyl groups (HAT—hydrogen atom transfer) and/or electrons (SET—single electron transfer) to free radicals, interrupting oxidation propagation and protecting the integrity of important biomolecules (e.g., DNA, lipids, proteins) against reactive oxygen and reactive nitrogen species. Phenolics bearing a catechol and/or a galloyl moiety can also act as secondary antioxidants by chelating transition metals, which are prooxidant agents [104]. In human physiology, phenolics can also stimulate the production of endogenous antioxidant defenses, such as the enzymes superoxide dismutase (SOD) and catalase, aiding in the reestablishment of a favorable balance between prooxidant and antioxidant factors. Although this mechanism has not been entirely unraveled, evidence suggests that the possible autoxidation of phenolic compounds triggers signaling pathways, leading to the production of antioxidant enzymes. In this case, even when not interacting directly with free radicals or prooxidant agents, phenolic compounds can still play a key role in the reduction in oxidative stress [105]. 

In legumes, gemination can affect the content of phenolic compounds, impacting their antioxidant activity. Pea and black bean sprouts germinated for 10 days showed an increase in total phenolic content (TPC). Bean sprouts’ content of phenolics went from 402.9 (day 0) to 634.1 mg of gallic acid equivalents (GAE)/100 g DW (day 10), reaching a TPC peak on day 6 (685.2 mg GAE/100 g DW). Similarly, pea sprouts increased from 584.3 mg GAE/100 g DW on day 0 to 850.5 mg GAE/100 g DW on day 10, reaching the highest TPC on day 7 (910.6 mg GAE/100 g DW). Consequently, the antioxidant activity of these legumes, measured as 2,2-diphenyl-1-picrylhydrazyl (DPPH) radical scavenging, oxygen radical absorbance capacity (ORAC), ferric reducing antioxidant power (FRAP), and Cu^2+^ chelating activity (CUPRAC), was enhanced with the increase in the germination period [106]. Germination increases the quantity of phenolic compounds due to the release of insoluble-bound phenolics, positively impacting the feedstock’s antioxidant activity. This effect has been demonstrated by Yeo and Shahidi [107] when evaluating the ratio of insoluble-bound to soluble phenolics in lentils during germination. Over four days, a significant increase in the amount of insoluble-bound phenolics was observed, leading to a bound/soluble ratio of 1.52 on day 4, as opposed to 1.43 on day 0. In another study using several soybean fractions [108], the concentration of isoflavones in the aglycone form was enhanced in the cotyledons and epicotyls after 144 and 168 h of germination, respectively. Other processes such as soaking, extrusion, fermentation, roasting, steam explosion, and high-pressure processing can have the same effect, as long as optimized conditions are used and minimization of phenolic degradation is achieved [109]. 

## 4. Bioefficiency of Bioactive Compounds of Legumes and Their By-Products 

The health benefits provided by legume bioactives is often limited by their bioaccessibility and bioavailability in the digestive system. Bioaccessible molecules are those efficiently released from the food matrix following oral ingestion, becoming available for absorption in the small intestine. In order to meet this requirement, the bonds between the molecule and other food components must be broken, which is often achieved by digestive enzymes in combination with the acidic and alkaline environments found along the gastrointestinal (GI) tract. In several instances, the bioaccessibility of conjugated bioactive compounds is impaired by the failure to break such bonds, as seen for insoluble-bound phenolics. The bioactives in the free form often demonstrate higher bioaccessibility than their bound counterparts due to the easier release from the matrix. Low solubility and poor stability in the GI tract are among other factors leading to the hindered bioaccessibility of several bioactive molecules [110,111]. 

Besides being released upon digestion, bioactives also need to cross the intestinal membrane and be absorbed. From this step on, these compounds and/or their metabolites can travel through the systemic circulation, reaching the target tissues where they can finally perform their biological actions. This characteristic is known as bioavailability. Bioactive compounds with high bioaccessibility have a higher chance of also displaying a satisfactory level of bioavailability, although some exceptions have been reported in the literature, especially for proanthocyanidins. Complex compounds, even when released in the small intestine, may encounter difficulties crossing the intestinal membrane due to their large molecular size and/or polarity differences. Thus, it is estimated that only 5–20% of polyphenols present in legumes can be absorbed in the small intestine, with the majority moving undigested to the colon [109].

The digestion of phenolic compounds begins in the oral cavity, where the food matrix starts to break down due to mastication. This exposes some food components to enzymatic hydrolysis performed by salivary α-amylase, with minor release of phenolic glycosides. Oral digestion is brief, with a minimal release of polyphenols. Phenolic digestion continues in the stomach, where the combined action of low pH (around 2.0) and enzymes such as pepsin and trypsin can efficiently release phenolic aglycones and those conjugated with proteins. Several studies of legume digestion indicate that the gastric phase is responsible for the highest release of phenolic compounds. During gastric digestion, some polyphenols can interact with pectin, reducing their biofficiency. A small fraction of phenolics is released in the small intestine (5–10%), a limiting step to their bioaccessibility. In this phase, free phenolics, including simple flavonoids and phenolic acids, demonstrate a higher likelihood of becoming available for intestinal absorption. Their transport mainly occurs by passive diffusion, moving to the liver where they can undergo sulfation, glucuronidation, and methylation. However, a majority of polyphenols in legumes (over 90%) move to the large intestine, including some soluble esters and glycosides, as well as all the insoluble-bound phenolics, where they are subjected to colonic microbiota fermentation. Besides releasing bound phenolics, fermentation can also yield phenolic metabolites, such as phloroglucinol aldehyde and phloroglucinol acid. Anthocyanins in the large intestine can undergo hydrolysis, demethylation, reduction, decarboxylation, dihydroxylation, and isomerization [110,111].

In the colonic environment, phenolic interaction with the gut microbiota is the key to gut health promotion. Polyphenols are able to modulate the microbiota, protecting the integrity of the mucosal barrier, attenuate inflammatory processes associated with colitis, and improve the epithelial barrier integrity, as reported for quercetin, daidzein, biochanin A, and formononetin, which are released after consumption of cooked chickpeas [112]. Phenolics can also prevent dysbiosis by reducing the colonic pH, favoring the growth of beneficial microorganisms, such as *Bifidobacterium* spp. and reducing the growth of pathogenic bacteria [109]. 

Other bioactive classes are affected in different ways by GI digestion causing challenges related to their bioefficiency. Bioactive peptides can have their intestinal absorption limited by their size, as peptide chain length determines their ability to cross the intestinal epithelial barrier. Short chain peptides can pass through active basolateral transport, whereas large size peptides are mediated by exocytotic vesicles. Additionally, peptide chains containing proline residues and phosphorylated amino acids can cause resistance to the action of digestive enzymes, hindering bioaccessibility. Likewise, digestion can be impaired by the degree of hydrophobicity. Bioactive peptides with a hydrophobic character can be more resistant to proteolysis [113]. 

Carotenoids, due to their lipophilic nature, follow digestion and absorption processes similar to those of dietary fats. Upon release from the food matrix, carotenoids are solubilized in oil droplets and transferred to bile salt, being further incorporated into micelles along with free fatty acids, mono- and diacylglycerols, phospholipids, and free cholesterol. This arrangement ensures the uptake of carotenoids by the epithelial cells in the small intestine’s luminal border. Protein receptors (e.g., scavenger receptor class B type I, cluster determinant 36) located in the apical membrane facilitate the transport of micelles. Passive diffusion of carotenoids across the brush border of epithelial cells is also believed to occur as a secondary transport mechanism. Inside the cells, carotenoids are incorporated into chylomicrons along with other dietary lipophilic substances and secreted across the basolateral membrane into the lymph. In the liver, carotenoids are released from chylomicron remnants and incorporated into very low-density lipoproteins (VLDLs) for distribution to peripheral tissues. Throughout the process, VLDL is converted to low-density lipoprotein (LDL), which continues to distribute carotenoids [114]. 

The key for carotenoid bioefficiency relies on the micellization process after food matrix release. This step will dictate the rate at which carotenoids will be absorbed in the enterocytes and further transported to the target tissues. The chromoplast (an organelle where carotenoids are accumulated) structure and the cell wall of plant material influence the release of carotenoids from the matrix and consequent micellization. Additionally, isomerization can deem some carotenoids more bioavailable. For instance, all-*trans*-lycopene is reportedly less bioavailable than *cis*-lycopene, being derived from heat-induced isomerization. The interaction between carotenoids and other food components can either enhance or hinder their bioefficiency. When co-consumed with dietary lipids, carotenoid micellization is increased as a result of higher solubilization and secretion of lipases, bile salts, and phospholipids into the intestinal lumen, thus positively affecting their bioavailability. Conversely, the intake of both carotenoids and fat-soluble vitamins may reduce carotenoids’ apical uptake into the enterocytes due to competition with the vitamins [114].

### Strategies to Increase the Bioefficiency of Legume Bioactives

**Figure 2:**
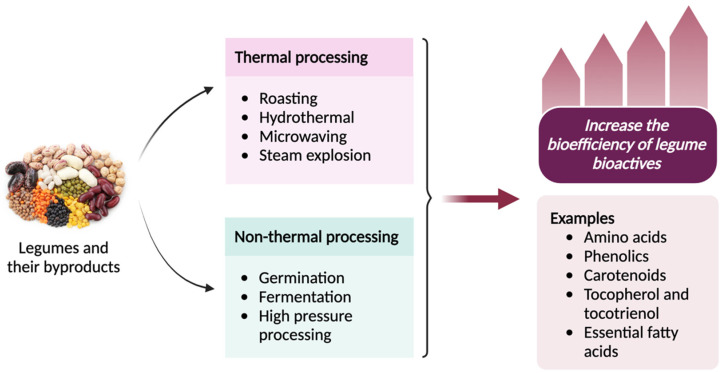
Thermal and non-thermal processing techniques used to increase the bioefficiency of legume bioactives. Created with Bio Render.

Increasing the bioefficiency of legume bioactives involves preserving them from environmental factors, improving their physicochemical functionalities and optimizing their absorption, utilization, and retention in the body. Several strategies can be used to enhance the bioefficiency of legume bioactives, such as both thermal and non-thermal processing as shown in Figure 2 [115]. Thermal processing can have a significant impact on the bioefficiency of legume proteins, influencing factors such as digestibility, amino acid profile, and overall nutritional quality. Cooking or heat processing can help reduce antinutritional factors such as protease inhibitors and lectins and improve protein digestibility [116]. Meanwhile, fermentation breaks down complex proteins, increases free amino acid content, and enhances bioavailability [117]. For instance, fermented legume products, such as temph or miso, have enhanced bioefficiency [118]. Fermentation and germination processes are found to improve the bioefficiency of fibers by breaking down complex carbohydrates and making them more digestible [119].

As discussed in Section 3.8, legumes are rich in phenolics as their major bioactive compounds. These molecules are present in the soluble and insoluble-bound forms. As already noted, insoluble-bound phenolics are covalently bound to cell wall cellulose, hemicellulose, pectin, and structural proteins. Therefore, they are not absorbed in the small intestine, which results in their low bioefficiency. Several studies have been carried out to release insoluble-bound phenolics from cell wall matrices and to enhance their bioefficiency using different types of food processing methods such as roasting, hydrothermal treatment, microwaving, germination, and fermentation [102]. Yeo and Shahidi [120] and Yeo et al. [121] revealed that hydrothermal (boiling) processing and fermentation of lentils released the insoluble-bound phenolics by disintegrating the cell wall matrix. However, the liberated bound phenolics were not efficiently converted into bioavailable soluble phenolics. 

Steam explosion is an emerging thermal processing method used in the food industry, where large cavities and intercellular spaces are created in the matrix, eventually breaking the cell walls. Cheng et al. [122] pre-treated adzuki beans with steam explosion at a pressure of 0.25–1.0 MPa for 30 s and 90 s. It was found that steam explosion enhanced the catechin, epicatechin, and protocatechuic acid contents in the free and bound phenolic fractions while increasing the antioxidant capacities of the phenolic compounds. Similarly, total phenolic contents in the free, esterified, glycosylated, and insoluble-bound phenolic fractions were increased upon steam explosion at 0.75 Mpa for 30 s by 1.47-, 1.87-, 1.73-, and 1.48-fold compared to untreated samples, respectively. In particular, the contents of ferulic acid, catechin, epicatechin, *p*-coumaric acid, and protocatechuic acid were enhanced upon steam explosion [123]. Therefore, steam explosion is one of the efficient methods to enhance the bioefficiency of bioactive phenolic compounds.

High-pressure processing (HPP), a non-thermal food processing technique, is used as a pre-treatment for traditional food processing to increase the biological activities and bioavailability of micronutrients and bioactive compounds. For instance, Redondo-Cuenca et al. [124] showed that HPP pre-treatment prior to traditional cooking improves the contents of bioactive compounds in four varieties of *Phaseolus coccineus* L. Moreover, high-pressure processed chickpeas exhibited enhanced total phenolic contents and antioxidant capacities measured by DPPH, ABTS, and ORAC assays compared to the conventionally cooked chickpeas [125]. 

Other non-thermal processing methods such as germination and fermentation have been found to increase the bioactive contents in dry beans. Mendoza-Sánchez et al. [126] used solid-state and liquid-state fermentation techniques with endogenous microbiota or *L. plantarum* to ferment kidney beans. It was found that beans fermented with *L. plantarum* increased the contents of bioactive compounds such as catechin, *p*-hydroxybenzoic acid, and feruloyl aldaric acid. It was also reported that the selection of appropriate microorganisms plays a major role in successful fermentation, which can hydrolyze the complex phenolic compounds into simpler forms by the proteolytic activity of bacterial enzymes during fermentation. On the other hand, germination also increased the concentration of phenolics, salicylic, coumaric, and caffeic acids, accompanied by an enhancement of their antioxidant activities in common beans (*Phaseolus vulgaris* L.).

## 5. Health Effects

**Figure 3:**
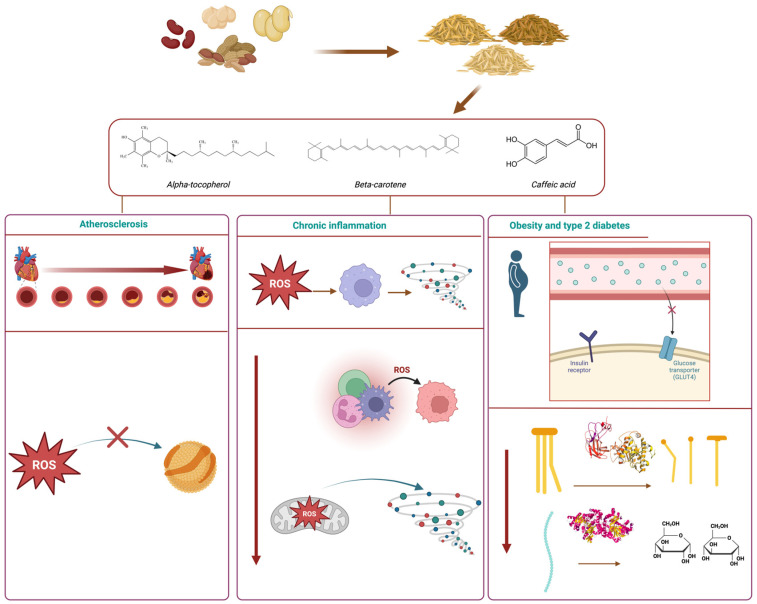
Main health benefits associated with the consumption of bioactive compounds contained in legume by-products. Created with Bio Render.

Figure 3 shows a schematic view of some of the health effects associated with bioactive compounds contained in legumes and their by-products.

### 5.1. Cardiovascular Diseases 

Several mechanisms are involved in the development and progression of cardiovascular ailments and intimately related to oxidative stress caused by excessive ROS accumulation. ROS can impair the function of endothelial cells lining blood vessels. Endothelial dysfunction is a critical early event in the development of atherosclerosis, the underlying cause of many cardiovascular diseases. ROS can damage the endothelium, disrupting its ability to regulate blood flow, maintain vascular tone, and contribute to the adhesion of inflammatory cells. Atherosclerosis is a condition characterized by the buildup of plaques in the arterial walls. The oxidation of LDL by ROS triggers inflammatory pathways and promotes the accumulation of oxidized LDL (Ox-LDL). As signaling pathways that promote the expression of adhesion molecules (e.g., macrophages) and proinflammatory cytokines are activated, Ox-LDL is taken up by macrophages, turning into foam cells. These adhesive cells can be deposited as atherosclerotic plaques, obstructing blood flow and forming atherosclerotic lesions, which can eventually lead to cardiac events, such as stroke and myocardial infarction [127].

Yeo and Shahidi [100] demonstrated that polyphenolic fractions of lentil hulls can suppress the formation of conjugated dienes, which are products from the oxidation of phospholipids, cholesterol esters, and triacylglycerols, which comprise human LDL-cholesterol. This bioactivity was demonstrated by both the soluble (18–23% of oxidation inhibition) and insoluble-bound (16–32% of oxidation inhibition) phenolics. On the other hand, no LDL oxidation protection was observed for dehulled lentils. Moreover, the polyphenolic fractions of lentil hulls have been demonstrated to act through the HAT and SET mechanisms, as evidenced by the 2,2-Diphenyl-1-picrylhydrazyl (DPPH) assay. Such outcomes show the importance of repurposing lentil by-products, as some of these fractions may compose the majority of the grain’s bioactive molecules. 

Two animal studies have shown that the consumption of bean paste and flour by mice ameliorated their lipid profile by reducing LDL and total cholesterol, as well as serum hepatic triacylglycerols [80,128]. Intervention studies using human subjects regarding legume consumption are scarce. In an 8-week randomized trial, 24 obese women consuming a calorie-restricted diet were divided into three subgroups (whole peanuts, peeled peanuts, and no peanuts). The group consuming 56 g of whole peanuts/day exhibited a body weight reduction of 3.2 kg, while the peeled peanuts group lost 2.6 kg. Both groups also showed a decline in their levels of total cholesterol and LDL-c, as opposed to the control group, which presented the lowest weight loss (1.8 kg) and no amelioration of their lipid profile [129]. A recent dose–response meta-analysis [130] concluded that a weekly intake of legumes, such as beans, lentils, peas, and chickpeas, at any level, is related to a reduced risk of cardiovascular and coronary heart disease, but not necessarily of stroke. The optimum legume consumption level to achieve cardioprotective effects appears to be around 400 g/week. 

### 5.2. Inflammation and Cancer

Inflammation is a defense mechanism of the body’s immune system, which can be triggered by the action of toxicants and/or pathogens. The inflammatory process involves the recognition of a stress factor by receptors located on the cell surface, leading to the activation of inflammatory pathways (e.g., mitogen-activated protein kinase, nuclear factor kappa-B), liberation of inflammation markers, and recruitment of inflammatory cells (leucocytes). Chronic inflammation results from a constant state of acute inflammation where the immune response lasts for a prolonged period and normal cell functions are compromised. Chronic inflammation may develop due to infections, autoimmune diseases, exposure to toxins, and oxidative stress, as well as smoking and other detrimental lifestyle factors. As such, this state underlies a plethora of health conditions, such as type 2 diabetes, cardiovascular ailments, and several types of cancer [131].

Green pea hulls, a rich source of phenolic acids and flavonoids, was used to produce polyphenolic extracts that were subsequently fed to Sprague Dawley rats, followed by biochemical analyses of the animals’ plasma and urine [20]. From 31 phenolic compounds found in the original pea hull extract, 10 were detected in the urine and blood of rats, along with 49 phenolic metabolites (mostly catechin, gallocatechin, kaempferol, quercetin, and their derivatives). The analyses of biomarkers showed that absorbed phenolics and their metabolites decreased the levels of plasma and tissue malondialdehyde (MDA), an indicator of lipid oxidation. Moreover, there was an upregulation of antioxidant enzymes, namely superoxide dismutase (SOD) and glutathione peroxidase (GSH-Px), which are key endogenous defenses against the onset of oxidative stress leading to chronic inflammation.

In another study, green pea hulls were subjected to in vitro simulated gastrointestinal digestion coupled with a Caco-2/RAW264.7 coculture model to investigate the anti-inflammatory effect of polyphenols from the digested material [23]. The small intestine was detected as the predominant site for phenolic release during pea hull digestion. Bioavailable phenolics exerted a significant anti-inflammatory effect on the cell model, reducing the secretion of nitric oxide (NO) and the proinflammatory cytokines interleukin-6 (IL-6) and tumor necrosis factor-α (TNF-α) by 50.9, 50.6, and 24.6%, respectively, compared to the control sample. The treatment was also able to inhibit the mRNA expression of the inflammatory mediators cyclooxygenase 2 (COX-2) and inducible nitric oxide synthase (iNOS) by 37.2 and 91.1%, respectively. Similarly, polyphenols in chickpea hulls stimulated the production of the antioxidant enzymes catalase and GSH-Px, while also downregulating the expression of nitric oxide and IL-6 on RAW 264.7 murine macrophage cells [131]. Proinflammatory cytokines act on recruiting and activating immune cells at the site of inflammation. Their overexpression contributes to the onset of chronic inflammation by dysregulating tissue repair mechanisms. Therefore, bioactives in residual fractions of legumes show promising therapeutic properties against common biomarkers of metabolic dysfunction.

Chronic inflammation is intricately linked to cancer. Besides immune system dysfunction, which impairs the body’s ability to recognize and eliminate cancerous cells, ROS generated by inflammatory processes can lead to DNA damage, depletion of DNA repair systems, and mutagenesis. Hydroxyl radicals (OH•) are particularly concerning due to their quick reaction rates. Some of the consequences of DNA attack by OH• radicals include base oxidation, and single-strand and double-strand scissions. Moreover, the overproduction of OH• radicals can damage DNA’s repair systems, leading to the replication of mutated cells. This mechanism underlies several cancer types, such as breast and pancreatic cancers [132].

Due to the anti-inflammatory properties of several phytochemicals present in legume by-products, these natural sources have also been investigated for their anticancer potential. Gazwi et al. [133] used lymphoma U937 and leukemic cells (THP1) to investigate the possible anticarcinogenic effect of pea seed coat extract. Although the extract’s active molecules were not identified in the study, cell proliferation was significantly reduced in a dose-dependent manner, with the concentration of 100 μg/mL showing the best outcome (cell proliferation decreased by 30% in comparison with the control). In another study [134], the presence of epigallocatechin and luteolin in extracts from colored pea seed coats demonstrated a strong correlation with their cytotoxicity toward cancerous cells (human colon adenocarcinoma LS174, human breast carcinoma MDA-MB-453, human lung carcinoma A594, and myelogenous leukemia K562). Caspase-3-dependent apoptosis and other apoptotic mechanisms were involved in the anticancer effect observed in LS174 cells.

### 5.3. Obesity and Type 2 Diabetes 

The impact of regular legume consumption in a balanced diet can be extended to potential lipid lowering effects, as well as inhibition of intestinal glucose transporters, α-glucosidase, α-amylase, and dipeptidyl peptidase IV, common biomarkers in the pathology of obesity and type 2 diabetes. Bioactive compounds, such as anthocyanins present in black beans, are believed to be responsible for these observed benefits and can act with a variety of mechanisms. Common beans (*Phaseolus vulgaris* L.) have been used for the production of bioactive peptides. Such peptides displayed in vitro antidiabetic activity by acting as inhibitors of α-amylase and α-glucosidase. Both enzymes catalyze the hydrolysis of complex carbohydrates, increasing glucose release into the bloodstream, which can lead to hyperglycemic events in diabetic patients. Phenolic compounds have also been extensively reported as inhibitors of α-amylase and α-glucosidase due to their ability to interact with these enzymes. Phenolic acids and flavonoids from lentils and black soybeans have demonstrated inhibitory activity toward α-amylase and α-glucosidase [135]. 

Protein hydrolysates obtained from the seeds of Bambara bean (*Vigna subterranean*) and Senegal coral bean (*Erythrina senegalensis*) display α-amylase and α-glucosidase inhibition with 50% maximal inhibitory concentration (IC_50_) of 0.256–12.12 and 0.314–16.14 mg/mL, respectively [136]. The best results corresponded to Bambara bean pepsin hydrolysate digested for 3 h (α-amylase inhibition) and Bambara bean trypsin hydrolysate digested for 5 h, both having a degree of hydrolysis of around 60%. Acarbose, a standard drug used as a control in the experiment, exhibited higher efficiency in inhibiting α-amylase (IC_50_ of 0.066) and α-glucosidase (IC_50_ of 0.166). Similar results were reported for anthocyanin-rich extracts of black bean by-products (seed coats, cotyledons, and sprouts) obtained by supercritical CO_2_ extraction [106]. It is important to note that due to acarbose’s inhibition mode (competitive inhibition), high-molecular-weight carbohydrates reach the colon undigested, resulting in intestinal discomfort caused by polysaccharide fermentation performed by the colonic microbiota. Protein hydrolysates and other bioactive compounds are procured as a natural alternative to this type of medication, although their mechanism of action and inhibition mode remain under investigation. The antidiabetic action of legume bioactives has not been extensively studied in animal models and human subjects, especially for legume by-products. Mojica et al. [137] investigated the in vivo bioactivities of hydrolyzed protein isolate from black beans using hyperglycemic rats. Protein isolates acted in a dose-dependent manner to block the transporters GLU2 and SGLT1, thus reducing glucose absorption.

The development of type 2 diabetes shows a strong correlation with overweight and obesity. The chronic imbalance between energy intake and expenditure, leading to a pathological expansion of the adipose tissue, characterizes obesity. As a multifactorial condition, obesity presents a high degree of complexity and its management and treatment require a plethora of interventions and drastic changes in the patient’s lifestyle. In this context, dietary changes are of upmost importance, with regular consumption of legumes and other whole foods playing a key role in the treatment of obesity and other metabolic conditions. Moreover, low-grade inflammation can be triggered by high body fat levels, leading to the activation of inflammatory signaling pathways in the adipose tissue [135].

Pancreatic lipase activity is a common biomarker for obesity due to the enzyme’s role in triacylglycerol hydrolysis and fatty acid absorption. Standard drugs, such as Orlistat, work by inhibiting pancreatic lipase, retarding lipid digestion. Nevertheless, common side effects associated with a continuous use of Orlistat include the decreased absorption of fat-soluble vitamins, flatulence, oily stools, and diarrhea. Bioactive compounds in legumes have been procured as alternative lipase inhibitors. For instance, polyphenolic extracts from Canadian cultivars of red and green lentils are rich in phenolic acids (e.g., *p*-coumaric and *p*-hydroxybenzoic acids) and flavonoids (e.g., kaempferol, quercetin, catechin glucoside, epicatechin gallate) and showed pancreatic lipase inhibition, with IC_50_ varying from 6.26 to 9.26 mg/mL [138]. A bioactive peptide (Cpe-III) from chickpea albumin demonstrated in vivo anti-obesity potential [139]. The peptide incorporated in the diet of hyperlipidemic mice significantly reduced serum total cholesterol and hepatic triacylglycerol levels, while increasing the activity of serum superoxide dismutase, an endogenous antioxidant enzyme. Moreover, molecular docking simulations showed that the Cpe-III peptide is able to bind the hydrophobic pocket of the cholesteryl ester transfer protein, inhibiting cholesterol transport, a promising feature in the development of nutraceuticals to control hyperlipidemia.

## 6. Legumes and Their By-Products as Ingredients in Functional Foods and Nutraceuticals 

Functional foods are products capable of providing health benefits beyond basic nutrition. Meanwhile, nutraceuticals are defined as “products derived from food but are used in medicinal forms such as pills, powders and potion not commonly associated with food” [140,141]. As evidenced by previous studies, legumes and their by-products are increasingly recognized for their potential in the development of functional foods and nutraceuticals, owning to their rich nutritional content and health-promoting properties. 

For instance, legumes are excellent sources of plant-based proteins, particularly soybeans, which are known for their high-quality protein, and contain all essential amino acids. Protein isolates and concentrates derived from these legumes can be used to fortify food products such as bakery goods, meat analogues, protein bars, and plant-based beverages and yogurt-like products [142]. Celiac disease is an inflammatory disease and autoimmune disorder caused by gluten consumption in bakery products. Therefore, replacing gluten in this type of food with other proteins capable of mimicking gluten’s viscoelastic property is necessary to provide suitable alternatives for people with celiac disease. It was reported that the incorporation of pea proteins and soy protein isolates in bread, cookies, cakes, and muffins could replace gluten [143]. Similarly, chickpeas, soybeans, peas, lupins, and lentil protein isolates can be used in food products to replace eggs, another allergenic food that is also associated with some metabolic diseases and high-cholesterol concerns [144]. Due to the cholesterol-lowering effect of legumes, they are valuable in the development of functional foods aimed at improving cardiovascular health. Legume-based dairy products, particularly soy milk, serve as alternatives for people with lactose intolerance [145]. Moreover, legume-based proteins can be used as encapsulation delivery systems for water- and fat-soluble bioactive compounds [146].

Isoflavones, particularly those in soybeans and other soy products, have gained attention due to their potential health benefits and their role in the development of functional foods, and have the ability to interact with estrogen receptors in the body. They can act as both estrogen agonists and antagonists, which may be beneficial in addressing hormonal imbalances [147]. This property makes isoflavones valuable in the development of functional foods targeted at menopausal symptoms and hormone-related conditions. In addition, they contribute to bone (prevent osteoporosis) and heart health (vascular function) [148].

Certain legumes, such as chickpeas and lentils, contain starches that resist digestion in the small intestine and reach the large intestine relatively intact. These starches can serve as prebiotics, promoting the growth of beneficial gut bacteria and contributing to gut health when incorporated into functional foods. In the large intestine, these starches undergo fermentation by gut microbiota and produce short chain fatty acids [149]. Furthermore, incorporating legume-derived oil, which is rich in α-linolenic acid, into food products can contribute to fortified omega-3 fatty acid content, offering potential cardiovascular benefits [150]. 

Besides the development of functional foods, bioactives from legumes, such as polyphenols, omega-3 fatty acids, and protein hydrolysates and isolates, can be encapsulated and developed as nutraceuticals in pills, powder, or other medicinal forms. In a systematic review, it was mentioned that soy isoflavone (genistein and daidzein) extracts in the form of nutraceuticals (supplements) could be used as a natural alternative for managing menopausal symptoms in individuals who either cannot or choose not to undergo hormone replacement therapy [151]. Pea fiber, obtained from the by-products of pea processing, can be encapsulated into nutraceutical supplements. These capsules could prevent hyperglycemia and protect gut microbiota against alterations induced by a high-fat diet [152].

Soybeans are a source of lecithin, a phospholipid known for its emulsifying properties. Nutraceutical supplements containing soy lecithin may be developed to support liver health and cognitive function [153]. Pea protein isolates or concentrates are commonly used in the development of protein powders. These nutraceutical products serve as plant-based protein supplements for individuals looking to increase their protein intake, especially those following a vegetarian or vegan diet [154]. Black soybean seed coats containing anthocyanins have been used in traditional oriental medicine for various health benefits. Thus, nutraceuticals in the form of black bean extracts or supplements may be developed to treat hypertension, diabetes, and cardiovascular diseases, potentially by reducing oxidative stress and inflammation [155,156]. 

## 7. Future Perspectives

Legumes, such as beans, peas, lentils, soybeans, chickpeas, and peanuts, are essential components of a balanced diet due to their rich macronutrient profile, which includes protein, carbohydrates, and essential fatty acids, as well as their composition of micronutrients and bioactive compounds. Although several legumes are staple foods in a great number of countries, their processing may lead to the loss of bioactive-rich fractions. By-products of legume processing, particularly hulls, are abundant in several bioactive classes, including polyphenols, carotenoids, tocopherols, and tocotrienols. Understanding the great variety of health-promoting substances contained in these discarded fractions may not only reduce agri-food waste generation, but also create new opportunities for the upcycling of legume by-products, leading to robust solutions for the growing functional food and nutraceutical sectors. For instance, legume proteins may be used as the source material for the production of bioactive peptides. The repurposing of legume residual fractions is of upmost importance from both environmental and economical standpoints. As sources of bioactive molecules, these by-products hold an excellent nutraceutical potential, which has been demonstrated by in vitro and in vivo evidence about their effects on chronic diseases, such as obesity, chronic inflammation, cardiovascular ailments, type 2 diabetes, and some cancers. In order to overcome the practical challenges associated with the use of legume residues for therapeutical purposes (typically low bioefficiency), conventional and non-conventional technologies can be employed, such as the pre-treatment of legume hulls with steam explosion and/or high-pressure processing to increase the bioaccessibility of bound phenolics. The ongoing climate crisis calls for a remodeling of the production chain, including the agri-food sector, which is responsible for a large proportion of waste generation. From farm to fork, feedstock needs to be fully utilized as a sustainable strategy to reduce the environmental burden associated with the majority of the current production systems. In this realm, legume by-products play a role in this new configuration by offering countless possibilities for their reuse, not only benefitting the environment but also taking advantage of the therapeutic potential of these fractions.

## Figures and Tables

**Table 1:** Phenolic composition of selected legume by-products.

Legume By-Product	Total Phenolic Content	Main Identified Compounds	Reference
Mung bean skin	105.4 μg/g DW (sum of individual phenolics in the insoluble-bound form)	Caffeic, ferulic, malic, quinic, sinapic, *p*-hydroxybenzoic, protocatechuic, isoferulic acids, and glycitin	[97]
Soybean hull	0.084 g of gallic acid equivalents (GAE)/100 g (free) and 0.015 g GAE/100 g (insoluble-bound)	Gallic, syringic, ferulic acids, (+)-catechin, (−)-epicatechin, quercetin, daidzein, genistein	[98]
Black lentil hull	40.26 mg GAE/g (soluble) and 40.96 mg GAE/g (insoluble-bound)	Formononetin, quercetin glucoside, quercetin, caffeic acid, *trans*-*p*-coumaric acid derivative (soluble). Myricetin, gallic acid, catechin, quercetin (insoluble-bound)	[99]
Green lentil hull	31.49 mg GAE/g (soluble) and 53.88 mg GAE/g (insoluble-bound)	Caffeic acid, *trans*-*p*-coumaric acid derivative, prodelphinidin dimer (soluble) Catechin, protocatechuic acid, myricetin (insoluble-bound)	
Green pea hull	6215.12 μg/g of extract (sum of individual phenolics in the soluble form)	Quercetin derivative, kaempferol trihexoside, epicatechin conjugate	[25]
Lentil hull	10.71–45.85 mg/g DW (sum of individual phenolics in the soluble form) 6.71–14.18 mg/g DW (sum of individual phenolics in the insoluble-form)	Coumaroyl glucoside, prodelphinidin dimer, catehin glucoside, luteolin-7-*O*-glucoside (soluble) Protocatechuic acid derivative, catechin, luteolin-7-*O*-glucoside (insoluble-bound)	[100]
